# Mechanism and bioinformatics analysis of the effect of berberine-enhanced fluconazole against drug-resistant *Candida albicans*

**DOI:** 10.1186/s12866-024-03334-0

**Published:** 2024-06-07

**Authors:** Sitong Wu, Wei Jia, Yu Lu, Hongkun Jiang, Chunlan Huang, Shifu Tang, Le Du

**Affiliations:** 1 Department of Laboratory Medicine, Key Laboratory of Precision Medicine for Viral Diseases, Guangxi Health Commission Key Laboratory of Clinical Biotechnology, Liuzhou People’s Hospital, Liu Zhou, 545006 China; 2 Ningxia Key Laboratory of Clinical and Pathogenic Microbiology, The General Hospital of Ningxia Medical University, Yinchuan, 750004 Ningxia China

**Keywords:** *Candida albicans*, Biofilm, Berberine, Fluconazole, Ergosterol

## Abstract

Biofilms produced by *Candida albicans* present a challenge in treatment with antifungal drug. Enhancing the sensitivity to fluconazole (FLC) is a reasonable method for treating FLC-resistant species. Moreover, several lines of evidence have demonstrated that berberine (BBR) can have antimicrobial effects. The aim of this study was to clarify the underlying mechanism of these effects. We conducted a comparative study of the inhibition of FLC-resistant strain growth by FLC treatment alone, BBR treatment alone, and the synergistic effect of combined FLC and BBR treatment. Twenty-four isolated strains showed distinct biofilm formation capabilities. The antifungal effect of combined FLC and BBR treatment in terms of the growth and biofilm formation of *Candida albicans* species was determined via checkerboard, time-kill, and fluorescence microscopy assays. The synergistic effect of BBR and FLC downregulated the expression of the efflux pump genes *CDR1* and *MDR*, the hyphal gene *HWP1*, and the adhesion gene *ALS3*; however, the gene expression of the transcriptional repressor *TUP1* was upregulated following treatment with this drug combination. Furthermore, the addition of BBR led to a marked reduction in cell surface hydrophobicity. To identify resistance-related genes and virulence factors through genome-wide sequencing analysis, we investigated the inhibition of related resistance gene expression by the combination of BBR and FLC, as well as the associated signaling pathways and metabolic pathways. The KEGG metabolic map showed that the metabolic genes in this strain are mainly involved in amino acid and carbon metabolism. The metabolic pathway map showed that several ergosterol (ERG) genes were involved in the synthesis of cell membrane sterols, which may be related to drug resistance. In this study, BBR + FLC combination treatment upregulated the expression of the *ERG1*, *ERG3, ERG4, ERG5, ERG24*, and *ERG25* genes and downregulated the expression of the *ERG6* and *ERG9* genes compared with fluconazole treatment alone (*p* < 0.05).

## Background

The infection rate of *Candida albicans* (*C. albicans*) has increased significantly, especially in immunocompromised patients and patients with organ intubation. According to statistics, systemic infection of *C. albicans* has a mortality rate of up to 31.8%, but the hospital intensive care unit is also the fourth highest risk factor for bloodstream infection [1, 2]. The rate of *C. albicans* infection was more than 50% in hospitals in the U.S., with a 40% mortality rate [3, 4]. The formation of biofilms can significantly reduce the sensitivity of fungi to azole drugs, resulting in drug resistance and sustained infection [5–7]. The formation of *C. albicans* biofilms can led to the production of extracellular matrix and a structure that encapsulates fungal cell populations, thereby hindering or delaying drug penetration; however, biofilms can also increase the expression of fungal efflux pump genes [8, 9]. FLC is a third-generation antifungal drug in the azole family that possesses a high degree of lipophilicity and low-toxicity and is a common first-line drug used to prevent and treat invasive *C. albicans* infections [10, 11]. In recent years, however, the formation of *C. albicans* biofilms has made the treatment of fungi ineffective, resulting in reduced of *C. albicans* susceptibility to fluconazole. This increase in drug resistance has hampered the penetration and absorption of azole drugs and reduced the number of antifungal drug targets [12–15]. In addition, mutations and the overexpression of *ERG11* weaken the binding of the drug to the target enzyme. The upregulated expression of the coding efflux pump genes *CDR1* and *CDR2*, which belong to the ATP family, and MDR can decrease the intracellular drug concentration in *C. albicans*, as well as the expression level of the gene *HWP1*, which encodes the mycelium cell wall-specific protein of *C. albicans* when the biofilm is formed [16–18]. At present, fluconazole can only inhibit the growth of fungal flora, not prevent it, and the emergence of fluconazole-resistant strains, as they can lead to repeated infections and hinder drug delivery, has presented a challenge. Accordingly, some researchers have aimed to identify drugs and treatment strategies that increase the sensitivity of *C. albicans* to fluconazole.

Berberine (BBR) is a common isoquinoline alkaloid found in many plants, particularly *Berberis vulgaris*. According to Ayurvedic and Chinese historical records dating back at least 3000 years, BBR was used to treat disease, with its medicinal properties having been reported to include significant antimicrobial activity against viral infections, fungi and inflammation. In terms of its clinical uses, BBR has been shown to help treat multiple diseases in vivo, such as tumors, diabetes, hyperlipidemia, and antiarrhythmic and immuno-suppressive diseases [19–22]. Recently, reports have also demonstrated that BBR, in combination with some antifungal agents, can be effective against *Staphylococcus aureus*, *Staphylococcus epidermidis*, *C. tropicalis*, and *C. glabrata* [23–26]. In this study, we found synergy between BBR and amphotericin B against *candidiasis* in vivo. To decrease the drug resistance and increase the sensitivity of *C. albicans* species, the ideal drug model was a combination of BBR and FLC, with FLC being one of the azole drugs used against *candidiasis* as a first-line treatment. Accordingly, the evaluation of such a drug model in vitro would contribute to the development of fungi-related therapeutics and targets.

In this study, we report that the molecular mechanism of BBR enhanced the susceptibility of *C. albicans* to FLC and reduced biofilm formation by altering the expression of efflux pump and adhesion genes, acting as a transcriptional repressor and altering cellular surface hydrophobicity (CSH), which are possible signaling pathways involved in drug resistance. In this study, we showed that the addition of BBR increased *C. albicans* susceptibility to FLC.

## Methods

### Strain cultivation and identification

The *SC5314* reference strain was used as the control; this strain was procured from the American Type Culture Collection. Thirty-six *C. albicans* strains that were isolated from the medical laboratory center of Ning Xia Medical University Hospital between January 2021 and June 2022 were used in this study and were inspected by an automated microbiological VITEK-2 COMPACT system. *C. albicans* species were collected from patient intubating catheters, stored at -80 ℃, and generally maintained in YPD medium (peptone 2%, dextrose 2%, and yeast extract 1%) at 30℃ in a rotatory shaker for 24 h [27]. The cells were washed three times in sterile PBS after centrifugation (2,000 × g, 15 min) and resuspended in RPMI 1640 at the required concentration before the experiment [28].

### Agents

**Fig. 1:**
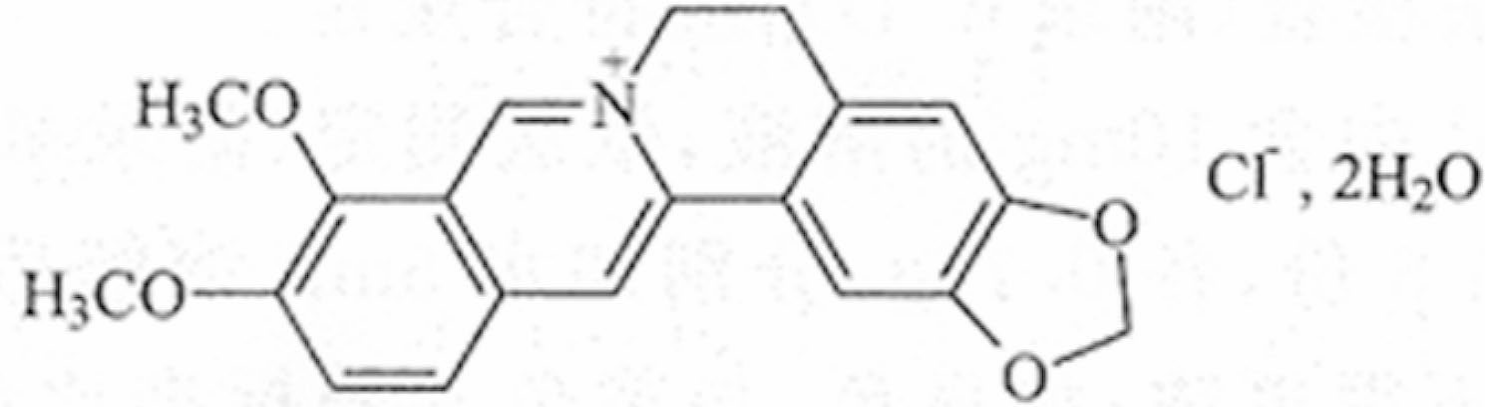
Molecular structure of BBR

Dimethyl sulfoxide (DMSO) solutions, including DMSO solutions of FLC and BBR (National Institutes Drug Control, Hang Zhou, China), were filtered to remove impurities, resulting in an initial concentration of 10,240 µg/ml. Sterile distilled water was then used to dilute the drugs to the desired concentration. The molecular structure of berberine is shown in Fig. 1.

### *Candida albicans* biofilm formation assay

Biofilm formation assays of *C. albicans* species were performed using 96-well microtiter polystyrene plates as stated previously [29]. Overnight cultures of *C. albicans* species underwent inoculation followed by shaking in YPD at 37 ℃ for 24 h. Subsequently, the bacteria were washed and resuspended in RPMI 1640 at a concentration of 1 × 10^6^ cells/ml for biofilm formation. Briefly, the experiment was implemented in 96-well microtiter polystyrene plates with 100 µL cell suspensions; the cells were subjected to a consecutive inoculation in a 5% CO2 atmosphere at 37 °C for 36 h or longer until mature biofilm formation was achieved without shaking. The nonadherent cells were washed in sterile PBS three times, then 200 µL methanol per well was added and the plates were left to dry for 15 min. The well plates then underwent crystal violet staining, with additional stain flushed under running water, and the biomass was quantified based on crystal violet OD570 nm absorbance.

### Susceptibility testing

The reference M44-A method is described by the Clinical and Laboratory Standards Institute (CLSI) [30–32]. Mueller Hinton agar (MH-MB, 90 mm in diameter) was complemented with 2% glucose and 0.5 mg/ml methylene blue. Sterile cotton swabs were utilized to adjust the turbidity of organisms in sterile physiological saline to a 0.5 McFarlane standard and the organisms were inoculated on the agar surface. The agar plate surface was covered with 25 µg FLC antifungal drug, and the plates were examined after 24 h of incubation at 37 ℃. The zones of inhibition were detected based on the CLSI criteria for in vitro testing. The results were described as zone diameters; ≤ 14 mm was considered resistant, ≥ 19 mm was considered susceptible, and 15 ~ 18 mm was considered close dependent SDD. We used the *SC5314* reference strain as the control strain.

### Checkerboard assay

The minimal inhibitory concentrations of *C. albicans* species were determined using the Clinical and Laboratory Standards Institute guidelines for M27-A2 via the broth microdilution method, as previously reported [33, 34]. Briefly, the fungal liquid was adjusted to 0.5 CFU/ml using a turbidimeter, the concentration was diluted 20 times and then 50 times, resulting in a final concentration of 2 × 10^3^ CFU/ml; an initial concentration of cell suspension (2 × 10^3^ CFU/ml, 100 µl) was added to 96-well microtiter plates. The test concentrations of BBR and FLC were in the range of 1024 to 0.25 µg/ml. After aerobic incubation for 24 h at 37 ℃, the cells were observed. The minimum inhibitory concentration of MIC60 was determined based on the absorbance at 630 nm. The interaction between BBR and FLC was evaluated using the checkerboard method, and the results are represented by the inhibitory concentration index (FICI), with FICI = (MICFLC in combination/MICFLC alone) + (MICBBR in combination/MICBBR alone); synergism was FICI ≤ 0.5, indifference was 0.5 ≤ FICI ≤ 4.0, and antagonism was FICI> 4.0.

### Time-kill curve test

In accordance with previous research [35, 36], a single larger colony of *C. albicans* cells in the exponential growth phase was picked from the medium. After washing with PBS three times, a standard turbidimeter was used to adjust the cell suspension to 15 × 10^4^ CFU/ml, which was then immediately diluted with RPMI 1640 to 5 × 10^4^ CFU/ml. The concentrations tested were 16 µg/ml FLC and 4 µg/ml BBR. The four experimental groups were the control group with no treatment, the FLC alone group, the BBR alone group, and the FLC + BBR combination treatment group. Samples from the experimental groups of *C. albicans* species were collected at predetermined time points (0, 4, 6, 8, 12, 24, 48 h), with aerobic agitation at 37 ℃ prior to counting. One milliliter samples of each solution were diluted 10 times, spotted on 1 ml YPD medium, and incubated at 37 ℃ for 24 h in a humid 5% CO2 atmosphere before colony counting. The experiment was repeated three times, and the CFU/ml was recorded. The combination treatment increased the killing by ≥ 2 log10 CFU/m compared to either drug alone, which was defined as synergy, while a killing effect within 2 log10 CFU/ml was defined as indifference.

### Fluorescence microscopy analysis

Briefly, a stock solution of fluorescein diacetate (FDA, Sigma) was prepared according to previously described methods [37, 38]. A concentration of 10 mg/ml FDA was dissolved in acetone and stock solution at -20 ℃ prior to use. A 1:50 working solution was freshly diluted with sterile PBS before each assay. Subsequently, the concentration of the fungal broth was adjusted to 1 × 10^6^ CFU/ml and incubated with 4 µg/ml BBR plus 16 µg/ml FLC for 4 h, 18 h, and 36 h at 37 ℃. In 96-well microtiter polystyrene plates, 100 µl of fungal liquid was added to 100 µl of FDA working solution. The microtiter polystyrene plates were placed on a shaking table for 30 min at 37 ℃ in the dark before being washed with unbound dye by sterile PBS and scanned under a fluorescence microscope at an emission wavelength of 518 nm and excitation wavelength of 494 nm.

### Cell surface hydrophobicity assay

The CSH of *C. albicans* species was tested using a water‒hydrocarbon two-phase assay [39]. Briefly, *C. albicans* species were collected in EP tubes, centrifuged at 3000 rpm for 10 min and then washed twice with PBS. Subsequently, the washed solution was removed to obtain 1 × 10^6^ cells/ml (OD_600_ = 1.0 in YPD medium). Then, 1.2 ml of cell suspension was moved into a clean glass tube and overlaid with 0.3 ml of octane. The cell suspension mixture was gently vortexed for 3 min and placed at 37 °C for 5-10 min for phase separation measurement at OD_600_ nm [40]. Each group was analyzed three times. The hydrophobicity was calculated using the following formula: [OD_600_ of control group - OD_600_ of test group)/OD_600_ of control group] × 100%.

### qRT‒PCR gene expression analysis

In a 24-well cell culture plate, 10% RPMI 1640 cell culture medium was added and the plates were incubated at 37 ℃ overnight and washed three times with sterile PBS solution. The concentration of the broth was adjusted to 2 × 10^6^ CFU/ml using a Mike turbidimeter, and 1 ml of the fungal suspension was added to each well of the 24-well plates; the plates were then placed in a constant-temperature shaker at 75 r/min and incubated at 37 ℃ for 2 h. The adhesion period of the yeast was followed by gently aspirating the suspension with a pipette and gently washing the wells two to three times with PBS. Then, 1 ml of RPMI 1640 cell culture medium was added and the plates were incubated at 37 ℃ 75 r/min for 24 h. During the maturation of the biofilms of the *C. albicans* species, the supernatant was gently removed with a pipette and the cells were washed twice to three times with PBS. After 36 h, the samples from four groups were collected: group A with 32 µg/ml FLC, group B with 8 µg/ml BBR, group C with 32 µg/ml FLC and 8 µg/ml BBR, and group D as the control group. For each group, RNA was extracted using an RNA purification kit (TaKaRa Ra Biotechnology). All the experimental steps took place on ice to prevent RNA or DNA degradation. First, cDNA was obtained using a reverse transcription kit (Takara Biotechnology). The thermal cycling conditions were as follows: an initial step at 94 ℃ for 5 min followed by 38 cycles and maintenance at 95 ℃ for 10 s, 58 ℃ for 30 s, and 72 ℃ for 30 s. After the reaction, the Light Cycler Real-Time PCR system was used to measure the CT of different genes. An endogenous reference control, 18 S RNA, was used. The primers of the *ALS3, CDR1*, *MDR*, *TUP1*, *HWP1*, and 18 S sequences are listed in Table 1. The gene levels were calculated using the following formula: 2 - (△△CT) [41]. The independent experiment was replicated three times.

**Table 1:** Sequences of primers used in the study

Gene	Primer sequences	Length
*ALS3-F*	CAACTTGGGTTATTGAAACAAAAACA	80
*ALS3-R*	AGAAACAGAAACCCAAGAACAACCT	
*ERG11-F*	AAGAATCCCTGAAACCAA	134
*ERG11-R*	CAGCAGCAGTATCCCATC	
*CDR1-F*	ACTCCTGCTACCGTGTTGTTATTG	192
*CDR1-R*	ACCTGGACCACTTGGAACATATTG	
*MDR-F*	GGTGCTGCTACTACTGCTTCTG	226
*MDR-R*	TGATGAAACCCAACACGGAACTAC	
*TUP1-F*	CACCCAGCACCAACAACGTT	330
*TUP1-R*	ATCTGTTGTTGTTGCTGCTG	
*HWP1-F*	TCAGCCTGATGACAATCCTC	105
*HWP1-R*	GCTGGAGTTGTTGGCTTTTC	
18 S-F	GGATTTACTGAAGACTAACTACTG	144
18 S-R	GAACAACAACCGCCCTAGT	

### Bioinformatics analysis

The biological significance of *C. albicans* species was investigated, and KEGG pathway enrichment analysis was used to analyze the pathways and functional annotations of different proteins. KEGG pathway and gene ontology (GO) analyses are common methods for functional systematic analyses of genes. These analyses were implemented for different proteins, with a p value less than 0.05 deemed significant for every comparison. The KEGG enrichment pathway analysis of the differently expressed genes between the groups and differentially methylated sites resulted in the identification of related genes, including *ERG1, ERG3, ERG4, ERG5, ERG6, ERG9*, and *ERG24.* The *ERG25* gene participates in *C. albicans* signaling pathways. The utilized primer sequences are listed in Table 2 [42–44].

**Table 2:** Sequences of primers used in the study

	Primer sequences	Length
ERG1-F	GCAACCGGCTGGTATCAAGGCA	183
ERG1-R	TCAACGGCATCAGGAACTGGCT	
ERG3-F	AAGATGGTGCTGTTCATG	157
ERG3-R	GGAATAGTTGCTGGGTTA	
ERG4-F	CTTCGGAAGGTCAATCTTGG	143
ERG4-R	GTCCAAACACCGGGTAAAGT	
ERG5-F	GCCGTAGCCAAAGCAACTGGC	117
ERG5-R	ACGGGGACCAGCAATTGAACCT	
ERG6-F	AGATGTTGGTTGGTGTAGGTG	235
ERG6-R	AACTGGAGCATGAACGGTAGC	
ERG9-F	TGGCCTCGAGAAATTTGG	163
ERG9-R	CAGTGACATGACCCAATGC	
ERG24-F	TCTTGGTGTTTGCCTACTGG	187
ERG24-R	GCTCTGCACTTCATTTCGTC	
ERG25-F	TGGATTGGCAGCAGAATATG	290
ERG25-R	TTTGGACCAGCTTCGGTATC	
18 S-F	GGATTTACTGAAGACTAACTACTG	144
18 S-R	GAACAACAACCGATCCCTAGT	

### Statistical analysis

All experiments were performed in triplicate on independent occasions. The results are displayed as the mean ± standard deviation, and *p* < 0.05 was regarded as statistically significant. The statistical analyses were performed using SPSS 22.0.

## Results

### Evaluation of the biofilm-producing capacity of clinical *Candida albicans* species

**Fig. 2:**
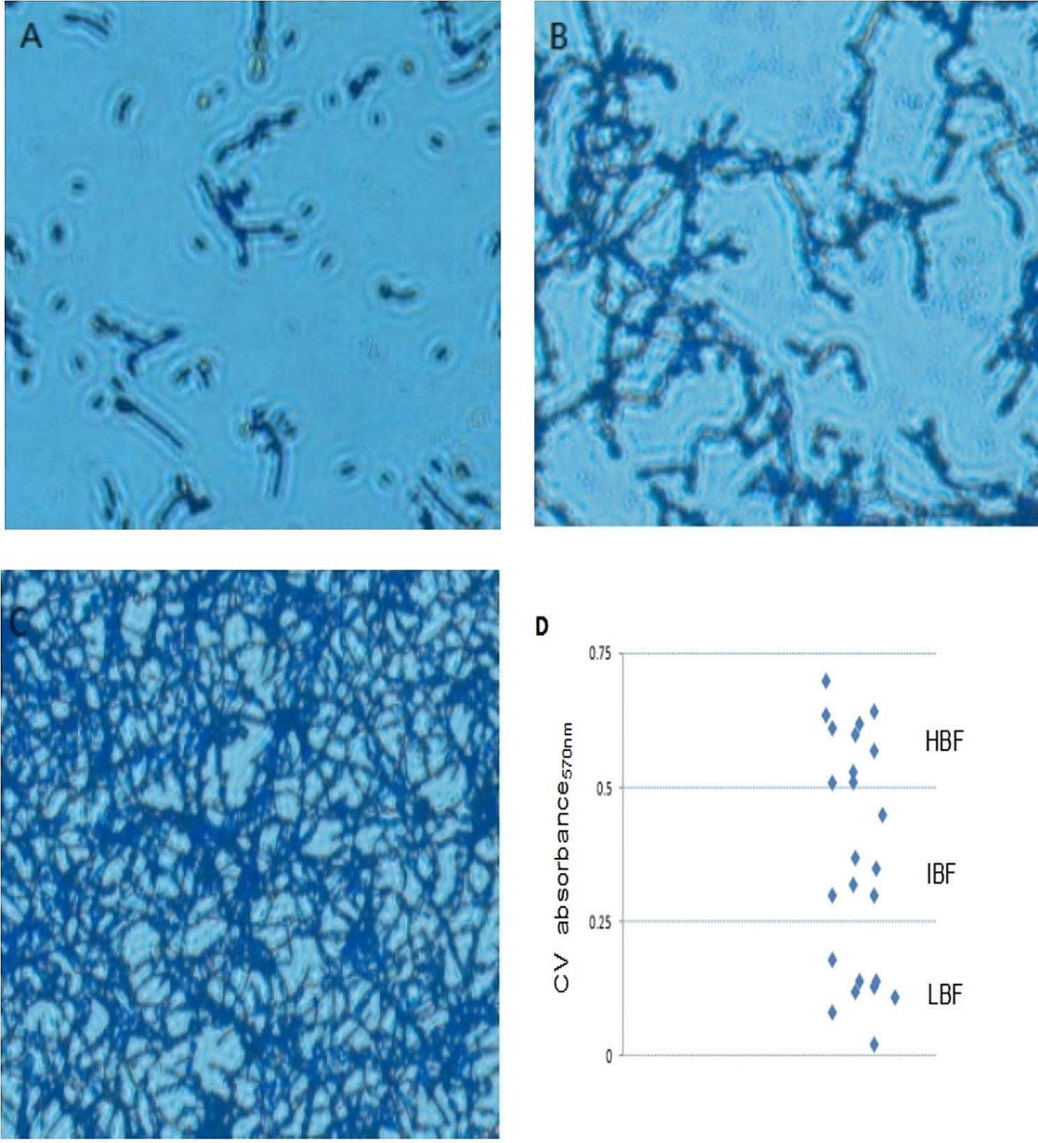
Strains with distinct biofilm formation capacities. *C. albicans* biofilms were isolated and cultured in 96-well plates for 48 h to produce a mature biofilm. The morphology of the biofilm was assessed via crystal violet staining and observed under a microscope. (**a**) A characteristic image of *C. albicans* with low biofilm formation (LBF). (**b**) A characteristic image of *C. albicans* with intermediate biofilm formation (IBF). (**c**) A characteristic image of strains with high biofilm formation (HBF). (**d**) Twenty-four strains of clinical *Candida albicans* strains were classified as LBF, IBF, or HBF strains

To assess the biofilm formation capacity of the clinical species, 24 species of *Candida albicans* analyzed via crystal violet staining. The results showed that the *C. albicans* species had distinct biofilm-producing capacities, which could be categorized as high biofilm formation (HBF), intermediate biofilm formation (IBF), or low biofilm formation (LBF) based on the absorbance at OD_570nm_. Eight clinical species fell into the LBF group with absorbance values (Q1 OD_570nm_ = 0.25) less than the first quartile, 10 clinical strains were in the HBF group with absorbance values greater than the second quartile (Q2 OD_570nm_ = 0.5), and six strains were categorized in the IBF group with OD_570nm_ values between the first and second quartiles Q1–Q2 (Fig. 2d). Based on macroscopic observation, the HBF strains had intact filamentous cells and a small number of yeast cells (Fig. 2c), while the LBF strains showed a lack of hyphal cells and a predominance of yeast cells (Fig. 2a). The species with capacities between the HBF and LBF strains were defined as IBF species, which had moderate filamentous phenotypes (Fig. 2b).

### In vitro antimicrobial susceptibility test

**Fig. 3:**
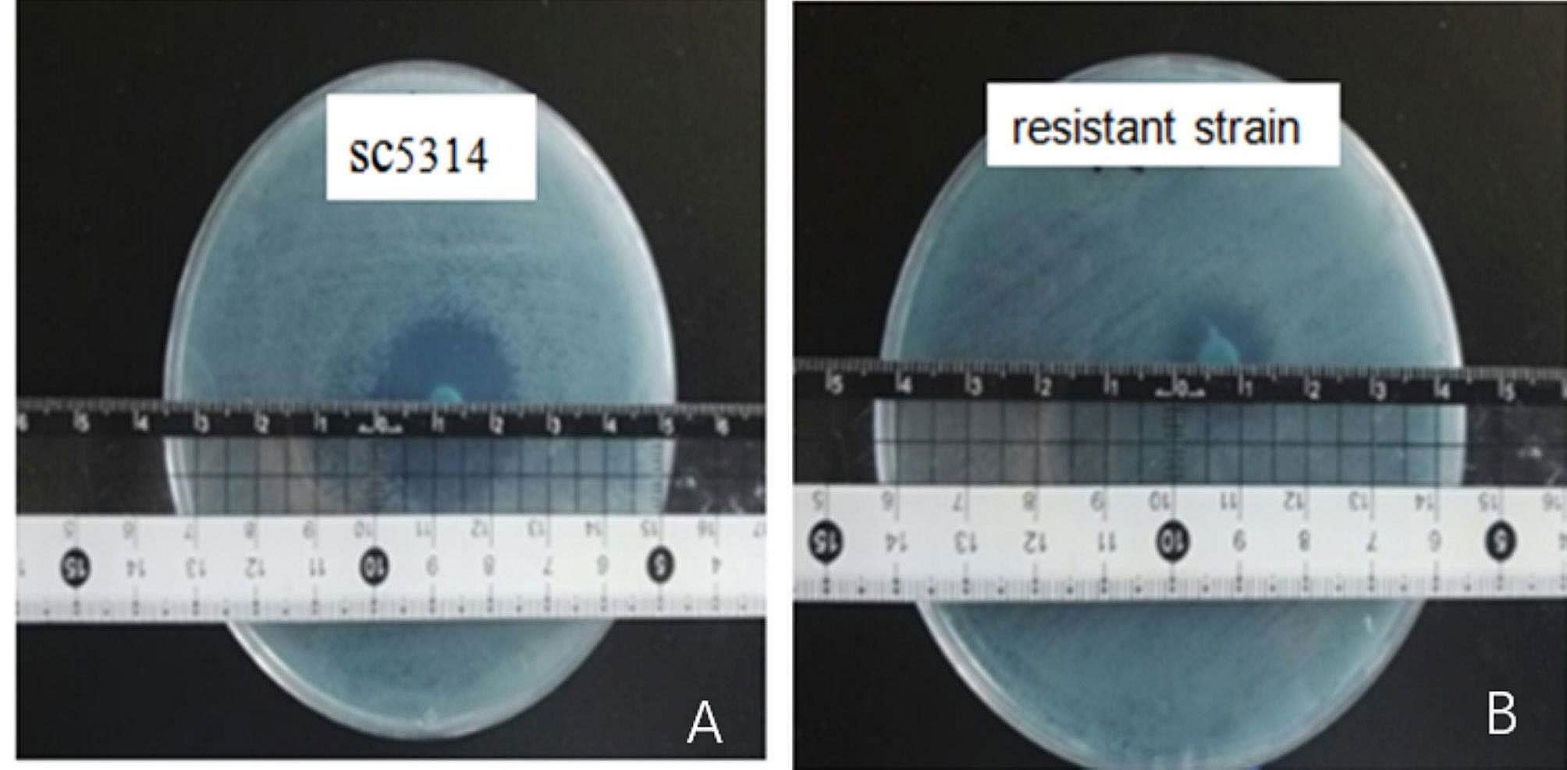
MH-HB agar plate assay evaluating the activity of FLC against the SC5314 reference strain and *C. albicans* isolates (resistant strains) with the agar plate containing 25 µg FLC. (**a**), (**b**) Images of the strains were taken after 24 h of incubation at 37 ℃. The diameter of the inhibition zone of the SC5314 reference strain was significantly larger than that of the *C. albicans* isolates

The sensitivity of *C. albicans* to fluconazole was determined by the Kirby-Bauer(K-B) method. The capacity of FLC to inhibit strain growth was determined by measuring the size of the diameter. The results showed that the *SC5314* reference strain and twenty *C. albicans* clinical strains were sensitive to FLC, with only two strains resistant to FLC. Among the library of pathogenic microorganisms, *C. albicans* had very little resistance to FLC; thus, the study of this fungus was considered to be of great significance. The susceptibility test results revealed that the diameter of the inhibition zone of the *SC5314* reference strains was 23 mm (Fig. 3a), and the diameter of the inhibition zone of the *C. albicans* species was 13 mm. Therefore, *C. albicans* species were selected as experimental strains (Fig. 3b).

### In vitro synergism of drugs against FLC-resistant *C. Albicans*

**Fig. 4:**
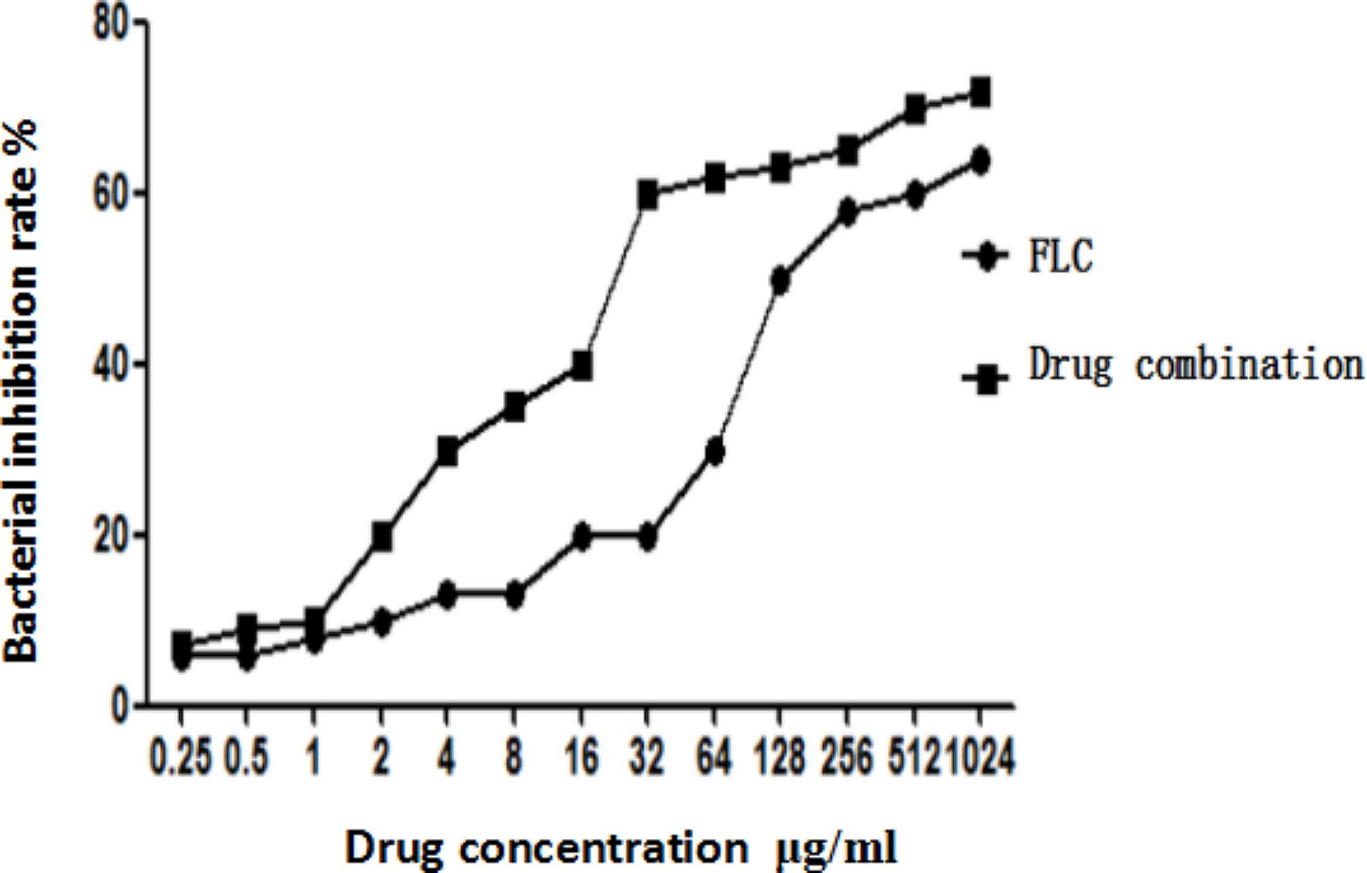
Concentration growth curves of *C. albicans* with different concentrations of FLC and drug combinations; these results demonstrated the antifungal effects of different concentrations

BBR is a natural isoquinoline alkaloid that is derived from coptis and phellodendron [45]. In this report, we evaluated the effect of the combination of FLC and BBR on the *SC5314* reference strain and *C. albicans* species via the checkerboard method, and the results are summarized in Table 3. When combining BBR and FLC treatment for the SC5314 reference strain, FLC only needed to be given at 4 µg/ml to achieve the MIC60 antifungal rate, and if FLC is used alone, 32 µg/ml was required. For *C. albicans* species, to reach the MIC60 antifungal rate, fluconazole only needed to be given at 32 µg/ml when used in combination, which is significantly lower than 512 µg/ml required when used alone, and the FICI < 0.5, which means that the two agents were synergistic (Table 3). Furthermore, FLC inhibition of *C. albicans* was dose dependent; this dose dependent activity was confirmed by a concentration growth curve assay. As shown in Fig. 4, the bacteriostatic effect of FLC alone was poor, and the rate of inhibition was still less than 40% when the concentration of FLC reached 64 µg/ml, but the addition of BBR improved the inhibition of *C. albicans* growth. Meanwhile, the dose of FLC and BBR used was decreased in the combination.

**Table 3:** MIC_60_ of FLC and BBR against different *C. albicans* strains (µg/ml)

Strain	FLC (µg/ml)			BBR (µg/ml)			Interpretation
	Alone	Combination		Alone	Combination	FICI	
*SC5314*	32		4	32	1	0.1563	Synergism
*C. albicans*	> 512		32	64	8	0.1875	Synergism

### Time-kill curves

**Fig. 5:**
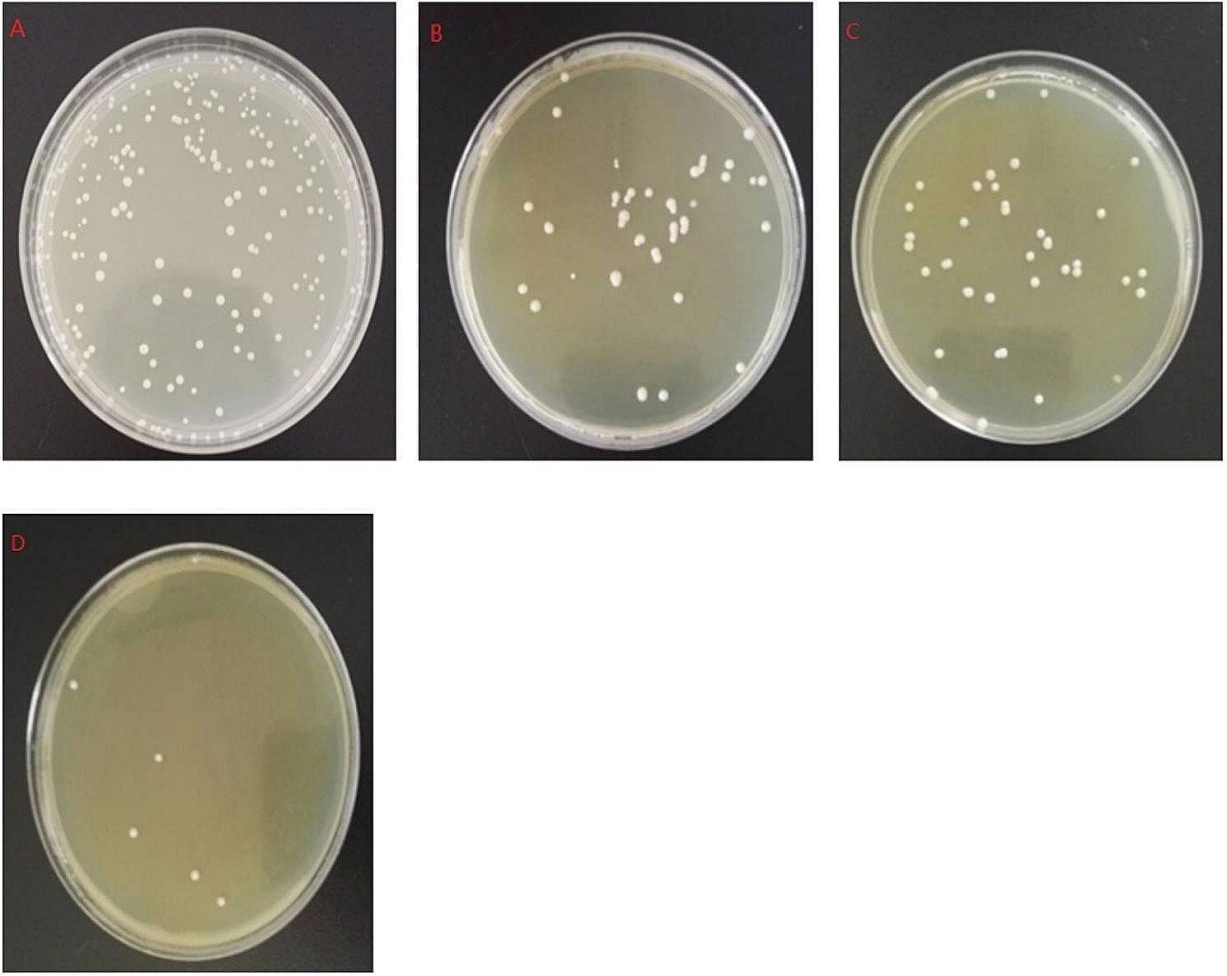
A combination of FLC and BBR inhibited the growth of the *SC5314* reference strain. (**a**) untreated group, (**b**) FLC 16 µg/ml treated group, (**c**) BBR 4 µg/ml treated group, and (**d**) combination of 16 µg/ml FLC and 4 µg/ml BBR. The strains were incubated at 37℃ for 6 h

**Fig. 6:**
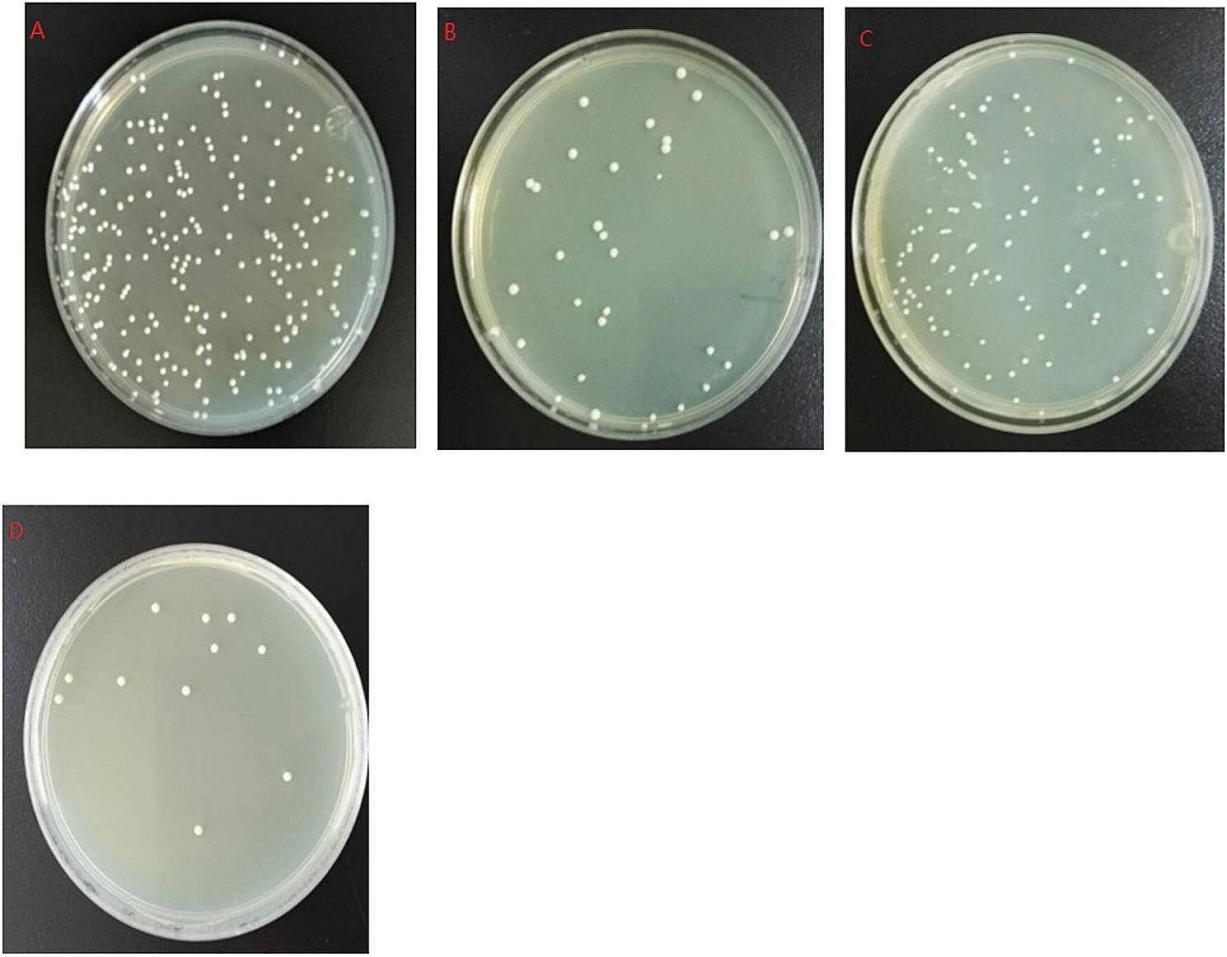
A combination of FLC and BBR inhibited the growth of *C. albicans* isolates. (**a**) untreated group, (**b**) FLC 16 µg/ml treated group, (**c**) BBR 4 µg/ml treated group, and (**d**) combination of 16 µg/ml FLC and 4 µg/ml BBR. The samples were incubated at 37℃ for 6 h

**Fig. 7:**
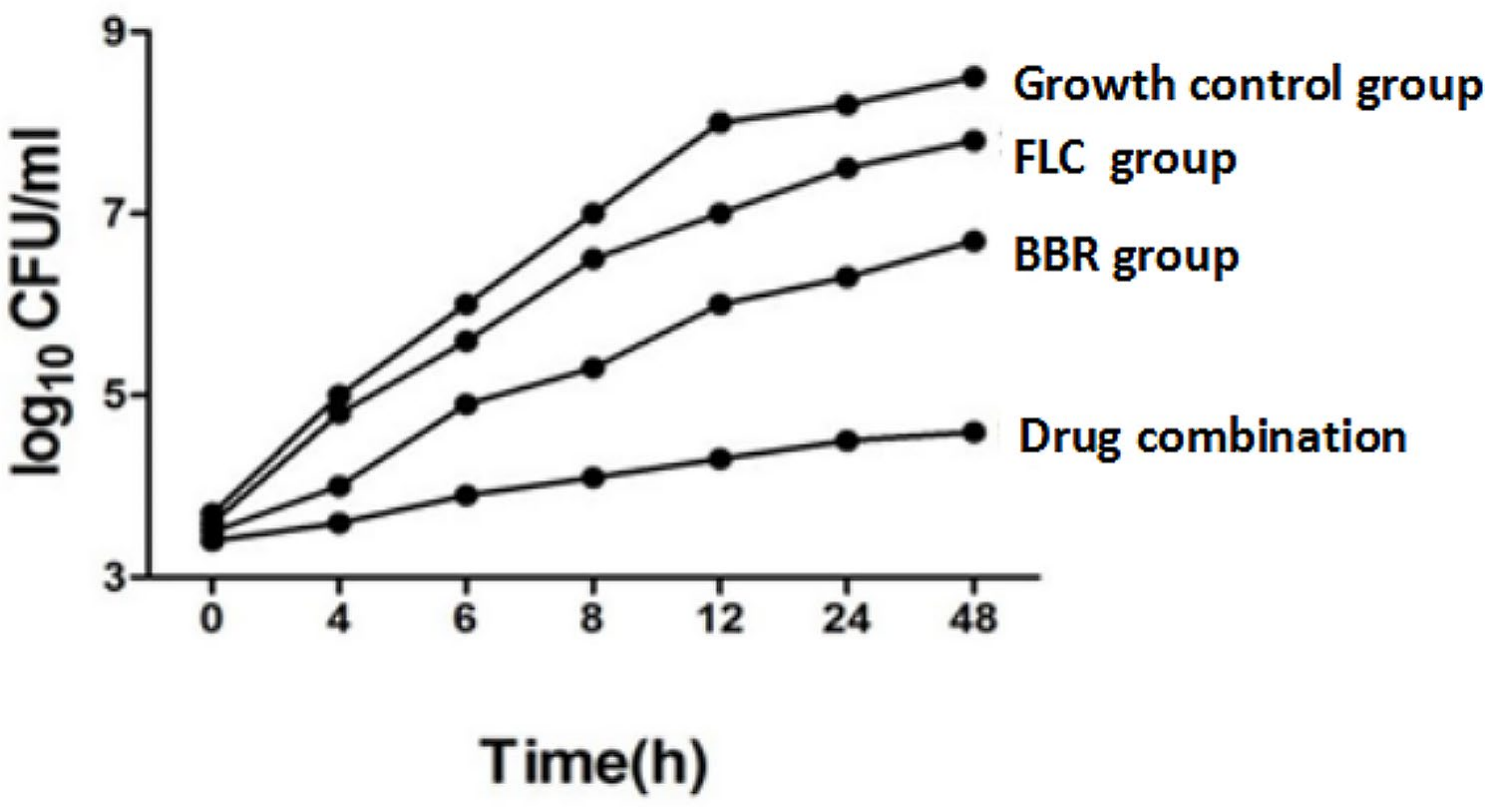
Time-kill curves of *C. albicans* isolates with the different treatments—control, FLC alone, BBR alone, and combination—using inoculums of 5 × 10^4^ CFU/ml. Aliquots were acquired at serial time points, and colonies were counted on agar plates after 48 h of incubation

To further investigate the dynamics of antifungal agents, we used time-kill curves, as they provided kinetic information on *C. albicans* growth and detailed information on the antifungal effects of the agents. The combination of FLC and BBR had a synergistic inhibitory effect on the growth of *C. albicans* species, and the growth was significantly different than that of those treated with single agents (*p* < 0.05) (Figs. 5 and 6). Time-kill curve analysis was performed according to a previous protocol and revealed the variation in *C. albicans* susceptibility to the different agents (Fig. 7). We found that 4 µg/ml BBR had a poor antifungal effect, while the addition of 16 µg/ml FLC had a stronger effect than BBR alone. The combination of the two agents led to a 2.1 log10 CFU/ml decrease in bacteria compared to that of FLC alone.

### The sensitivity of *C. Albicans* to FLC increased after BBR treatment

**Fig. 8:**
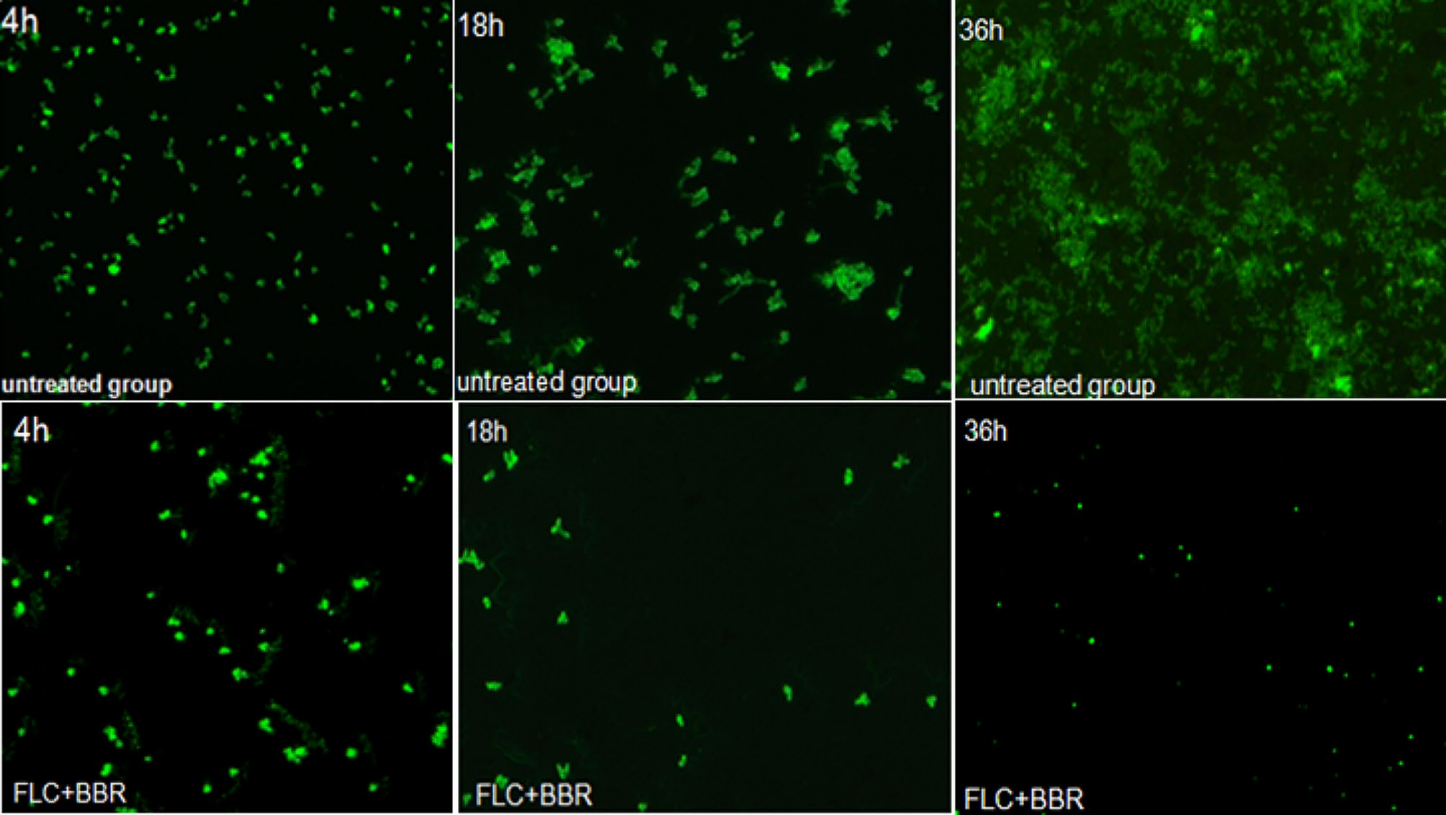
*C. albicans* isolates were cultured with 16 µg/ml FLC and 4 µg/ml BBR for 36 h. The inhibition of *C. albicans* growth was evaluated by fluorescence microscopy. BBR significantly increased FLC-induced inhibition of the growth of biofilm-producing C. albicans isolates after 36 h of incubation, and a combination of 16 µg/mL FLC and 4 µg/mL BBR dramatically inhibited cell growth and biofilm formation compared with the control treatment (*p* < 0.05)

We used a fluorescence microscope to observe the morphological changes in *C. albicans* species after exposure to the combination of BBR and FLC (16 µg/ml FLC and 4 µg/ml BBR). BBR has been shown to enhance the susceptibility of *C. albicans* species to FLC. The addition of 4 µg/ml BBR with FLC dramatically increased the susceptibility of biofilm-producing *C. albicans* species to FLC. Morphological analysis using fluorescence microscopy further showed that a combination of 16 µg/ml FLC and 4 µg/ml BBR inhibited *C. albicans* growth and hyphal formation (Fig. 8).

### BBR and FLC synergistically decreased the hydrophobicity of C. Albicans

**Fig. 9:**
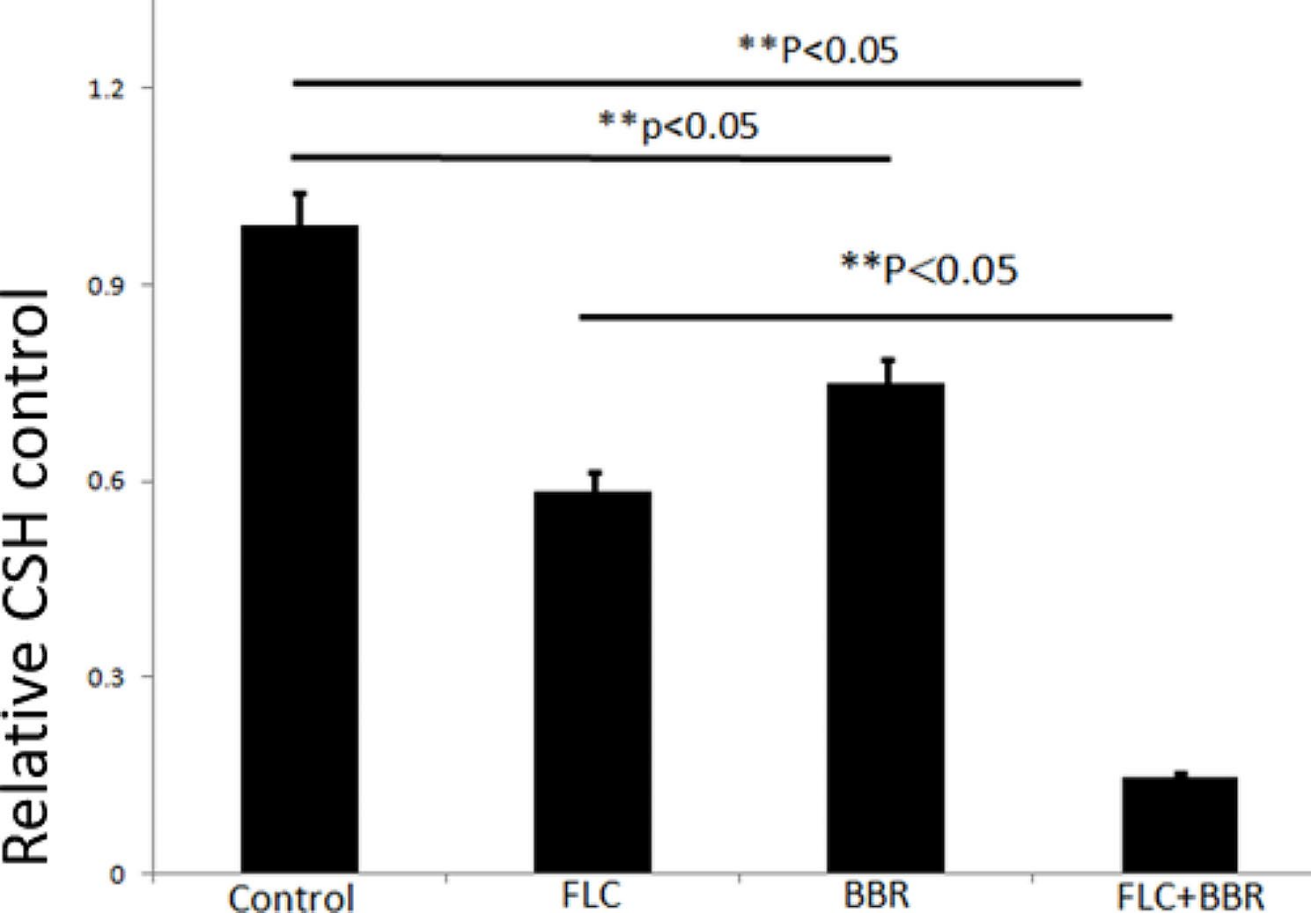
Comparison of cell hydrophobicity in the different treatment groups. The agents synergistically decreased the CSH of *C. albicans* isolates. *C. albicans* isolates were cultured in the presence of 32 µg/ml FLC or 8 µg/ml BBR alone or a combination of BBR and FLC. The BBR and FLC combination led to a significant decrease in the CSH of the *C. albicans* isolates, although BBR and FLC alone also moderately reduced the CSH when compared to the untreated control, BBR alone, and FLC alone groups (*p* < 0.05)

The effect of the different treatments on cell hydrophobicity was determined based on the principle of water‒hydrocarbon two-phase separation. The measurement results showed that when FLC alone was added at a concentration of 32 µg/ml or BBR alone was added at 8 µg/ml, the CSH was reduced when compared with that of the untreated group (*p* < 0.05). When the agents were used in combination, the decrease in CSH decrease was obvious and the was significant when compared with that of FLC or BBR alone (*p* < 0.05), as shown in Fig. 9. The results revealed that the combination of FLC and BBR synergistically decreased the cell surface hydrophobicity of *C. albicans* species.

### The combination of FLC and BBR reduced the expression of drug resistance genes

**Fig. 10:**
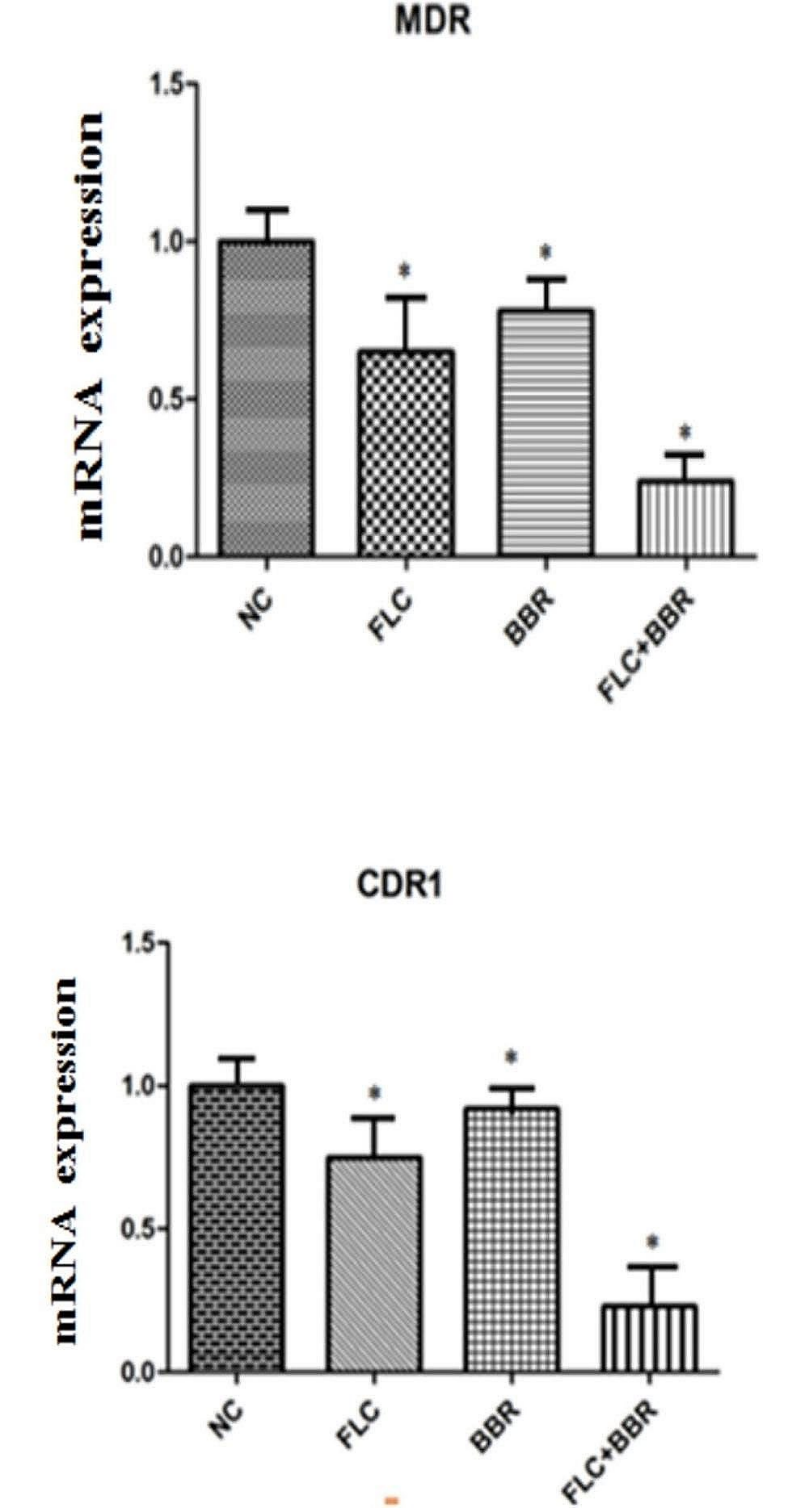
The mRNA expression levels of *CDR1*, *MDR*, *ALS3*, *ERG11*, *TUP1*, and *HWP1* in the different drug treatment groups were determined via qRT‒PCR, *p < 0.05

To investigate the mechanism of the effects of the FLC and BBR combinations on *C. albicans* species, we further investigated the underlying molecular mechanism involved in increasing the sensitivity of biofilm-producing *C. albicans* species to combination treatment; the overexpression of genes is one of the usual mechanisms of drug resistance in *C. albicans* species, such as those encoding drug targets such as ERG11, the efflux pump genes *CDR1* and *MDR*, the adhesion function gene *ALS3*, and the hypha-related gene *HWP1*. The transcription of the *TUP1* virulence genes in the cells under 32 µg/ml FLC and 8 µg/ml BBR treatment was measured by qRT‒PCR assay. Consistent with previous reports, our results showed that FLC and BBR combination treatment, which was remarkably different when compared with treatment with the same agents alone (*p* < 0.05), inhibited the expression of *CDR1*, *MDR*, *ALS3*, and *HWP1*. Of note, both FLC, BBR, and the synergistic combination treatment failed to inhibit *TUP1* gene expression in the clinical *C. albicans* species (Fig. 10).

### DNA extraction, genome sequencing, assembly, and annotation

The genomes of the *C. albicans* species were sequenced using the Illumina HiSeq2000 platform. The final assembly included 15,445,198 bp and consisted of 72 scaffolds and 72 contigs. The overall GC content of the genome was 35.13% (Table 4). The data generated with the PACBIO platform were corrected to the generated assembly sequence by the Illumina platform to alleviate the sequencing error rate caused by the relatively high pressure. The average gene length was 1435 bp. The number of tRNA, SnRNA genes, and microsatellite sequences (144, 32, and 2384, respectively) identified in the assembly were > 2X that of other Candida sp.

**Table 4:** Features of *Candida albicans* genomes

Feature	Candida albicans
Genome size (bp) 15,445,198	
GC content (%)	35.13
Gene number (#)	6276
Gene average length (bp)	1435
Gene internal length	643,853
Gene internal GC content	31.29
Gene length	9,006,661
% of genome (Genes)	58.31
% of genome (Internal)	41.69
tRNA number (#)	144
snRNA number (#)	32
TRF	4415
Minisatellite DNA	2384
Microsatellite DNA	1362

**Fig. 11:**
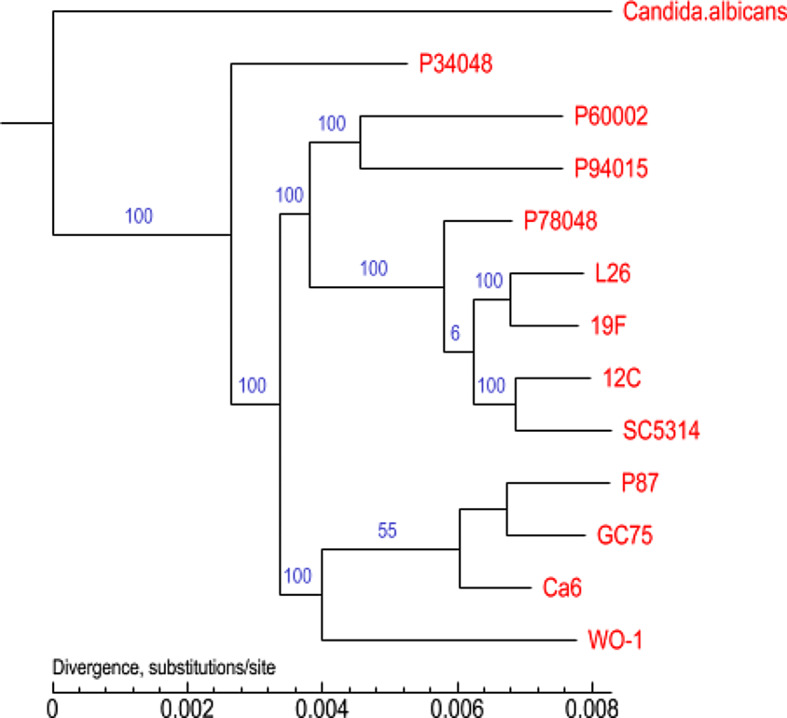
Significantly enriched gene ontology categories with the classification of differentially expressed genes. The results are summarized in the following three categories: molecular function, cellular component, and biological process

**Fig. 12:**
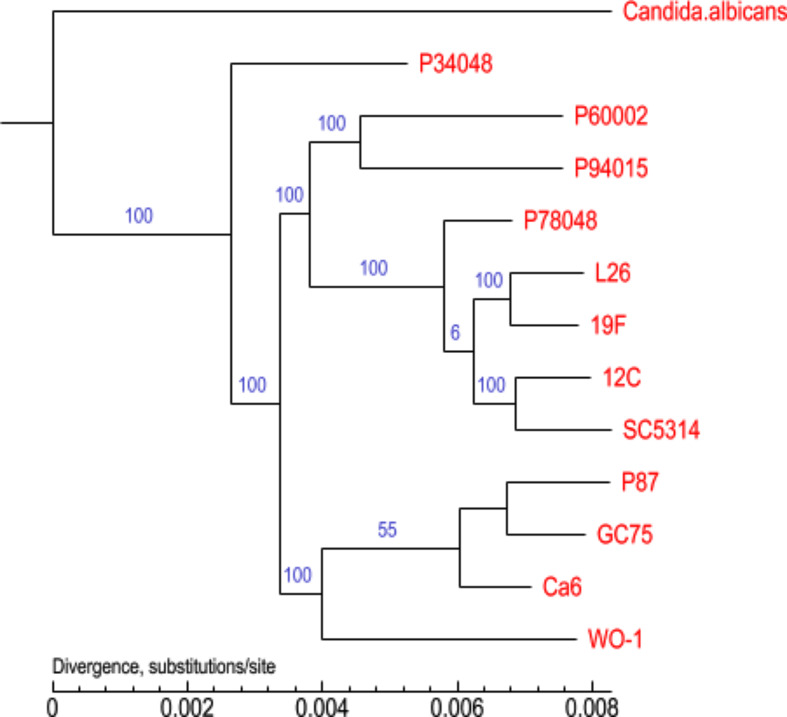
KEGG pathway enrichment analysis results in *C. albicans* isolates; multiple metabolic pathways were highly enriched. The X-axis indicates the name of the pathway; the Y-axis denotes the number of genes

In total, 6276 protein-coding genes were predicted with a total length of 9,006,661 bp in the genomes of the *C. albicans* species. Additionally, the VFDB database predicted that the *C. albicans* species had 763 virulence-related genes involved in the formation and regulation of toxins and invasive enzymes. The CARD database annotation showed 82 related resistance genes were associated with *C. albicans*. GO terms were assigned to the 14,972 protein-coding genes of the *C. albicans* species. Almost 14,972 genes were assigned to each of the following categories: molecular function, cellular components, and biological process (Fig. 11). A total of 8149 genes participating in 40 pathways were annotated using the KEGG database (Fig. 12). Six of these pathways were significantly enriched, including cellular processes, environmental information processing, genetic information processing, human diseases, and metabolism in organismal systems. KEGG analysis was implemented to categorize the functions of the identified genes, with the amino acid metabolic pathway revealed to be the most significantly enriched pathway; the metabolic genes of the *C. albicans* species were mainly involved in acid and carbon metabolism. These results indicate that these two types of genes account for a significant proportion of the biological processes involved in Candida growth, death, signal transduction, and translation. This result may explain how *C. albicans* acquires drug resistance as a conditional pathogen.

**Fig. 13:**
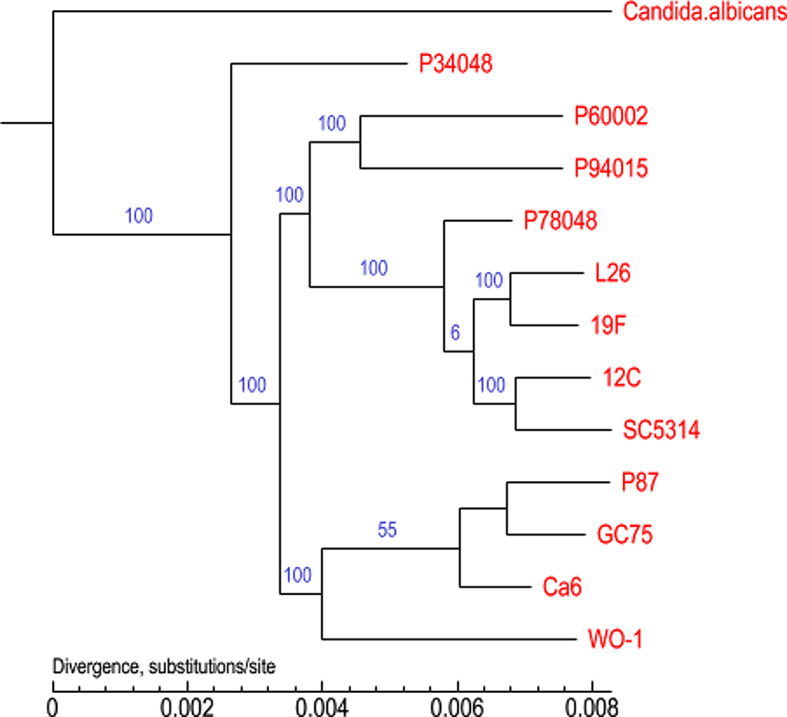
A phylogenomic tree was constructed based on selected proteins. Phylogenetic relationships among 13 species are shown. The numbers indicate the branch lengths

The phylogenetic tree of the 13 *C. albicans* species was constructed using homologous protein analysis (Fig. 13). In the phylogenetic tree, nodes represent the closest common ancestor of each branched strain, and the length of the line segment between the nodes represents the evolutionary distance of each strain. As seen from the figure, in this study, we showed that the *C. albicans* species were closely related to P34048, which is from the same family, but the *SC5314*, *Ca6*, and other strains were distant. The collinearity analysis showed that the collinearity of the nucleic acids between the *C. albicans* species and *SC5314* was distinctive. Phylogenetic analysis and the GO database were used to further analyze the gene information to extract effective information from the large amount of gene data; this information could provide valuable clues for future research.

### Gene duplication and collinearity analysis

**Fig. 14:**
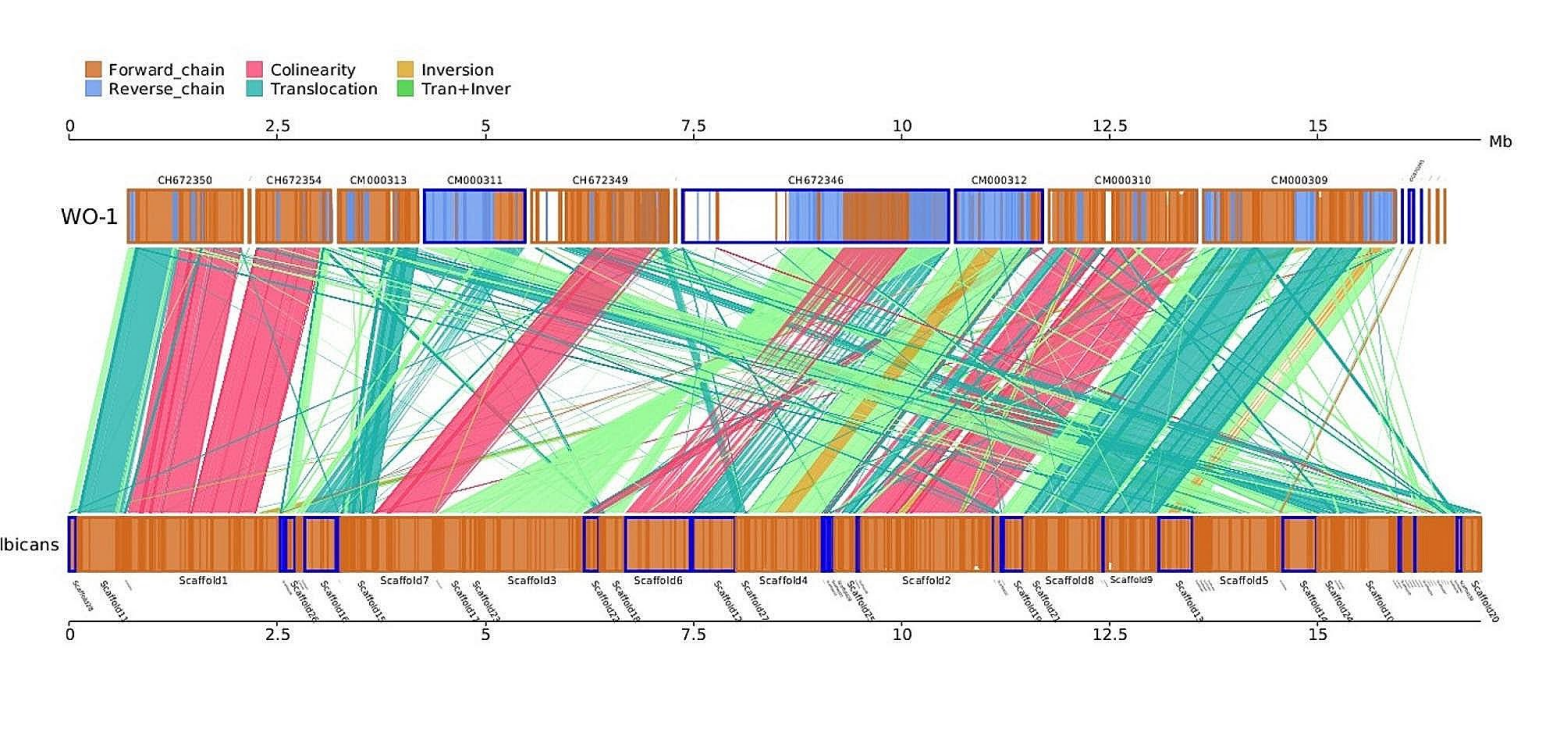
The yellow box shows forward genome chaining; the blue box shows reverse genome chaining. The width of the fill color in the box indicates the similarity of the alignment, with a full fill indicating a 100% similarity

The interspecific synteny among the *WO-1, SC5314*, and *P34048* strains and the *C. albicans* species were analyzed and compared to further explore the evolution of the resistance genes. Interestingly, the *C. albicans* species and P3408 showed a highly similar collinear relationship (Fig. 14). However, the gene correspondence between the *C. albicans* species and the *SC5314* and *WO-1* strains was poor. These results are consistent with what was seen in the phylogenetic tree. The figures show the structural variation that occurs in the process of evolution, such as the insertion and deletion of sequences and the genetic changes caused by transposition, recombination, and other mechanisms.

### Expression of genes in the steroid biosynthesis pathway

**Fig. 15:**
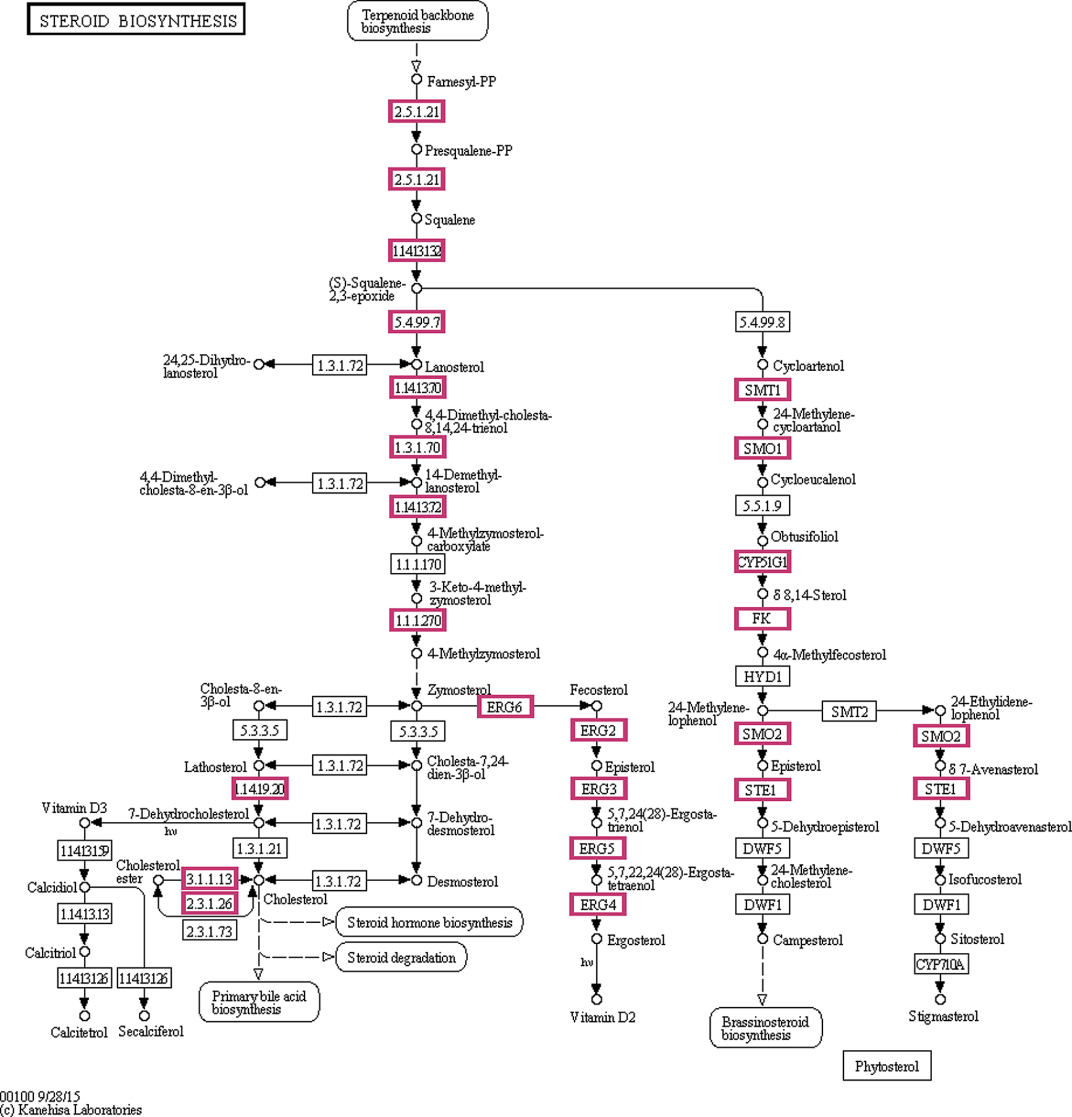
The steroid biosynthesis and transformation pathway proceeds from terpenoid backbone biosynthesis to the end products in *C. albicans*. The pathway shows the common ergosterol synthesis process with enzymes and key genes. The metabolites in red indicate the genes that had upregulated expression in the steroid biosynthesis pathway

**Fig. 16:**
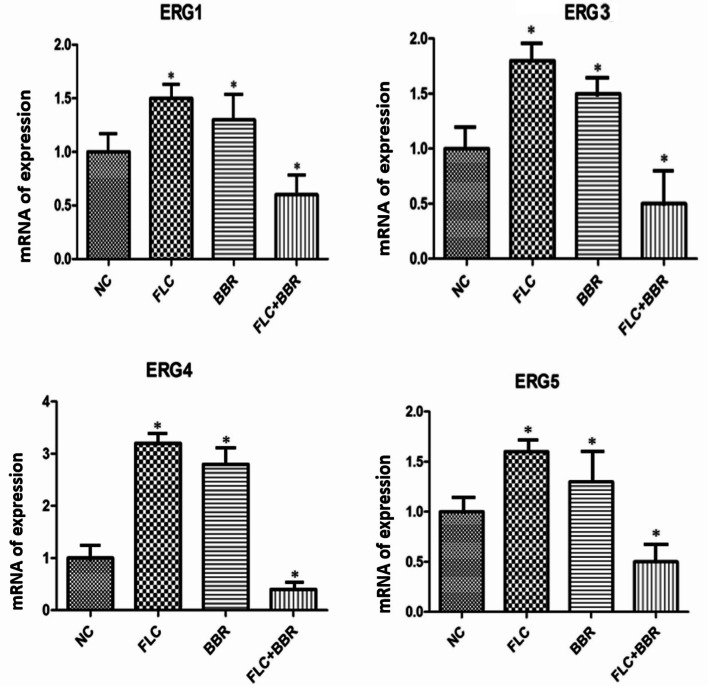
Gene expression in *C. albicans* after BBR and FLC treatment. *C. albicans* isolates were treated with 32 µg/ml FLC and 8 µg/ml BBR, 32 µg/ml FLC alone, or 8 µg/ml BBR alone; the control group was not treated. Three independent experiments were performed, with 8 replicates in each group

Figure 15 shows the steroid biosynthesis pathway, where genes marked in red had upregulated expression. Ergosterol is a key target gene in cell membrane synthesis and a therapeutic target for antifungal drugs. Most antifungal drugs counter the synthesis of steroids by inhibiting the activity of genes in the ERG family. Compared with the control group, *ERG1*, *ERG3*, *ERG4*, *ERG5*, *ERG24*, *and ERG25* expression in the FLC alone or BBR alone group was upregulated; however, in the FLC and BBR groups, there was significantly downregulated expression of these genes compared with the other groups (*P* < 0.05) (Fig. 16). No significant difference was detected between the FLC or BBR alone treatment groups. *ERG6* and *ERG9* expression was significantly increased in the FLC and BBR groups compared to the FLC alone or BBR alone groups (*P* < 0.05). The figure shows that the combination of BBR and FLC increased drug sensitivity by regulating the expression of genes involved in intracellular mechanisms in *Candida albicans*.

## Discussion

*Candida albicans* is an opportunistic pathogen that often infects immunocompromised patients [5]. Although FLC agents are used to prevent *Candida albicans* infections, the emergence of drug resistance remains a clinical challenge. With the increasing resistance of *Candida albicans* to FLC, an increasing number of experiments have focused on the development of new antifungal drugs. In this regard, we investigated possible risk factors, including biofilm formation. While the molecular mechanism of *Candida albicans* resistance to FLC has been extensively researched in the past few years, to the best of our knowledge, there is no effective treatment for *Candida albicans* infections in clinical settings [46, 47]. An increasing amount of evidence has suggested that *Candida albicans* biofilms could accelerate resistance to antifungal agents. Accordingly, studies with the aim of identifying potential antifungal drugs or targets to prevent further drug-resistance are urgently needed.

In recent years, several studies have reported that a combination of BBR and antibiotics could synergistically increase the susceptibility of *Candida albicans* to the antifungal FLC [26]. Previous studies have shown that BBR can induce DNA damage [48]. In this study, our results demonstrated that BBR can increase the sensitivity of *Candida albicans* by inhibiting biofilm and hyphal formation, drug transportation, and cell adhesion while simultaneously decreasing hydrophobicity and increasing the concentration of drugs entering the cell.

The biofilm of *Candida albicans* is composed of a mixture of yeast cells and mycelium, and the formation of biofilms is important for drug resistance [49, 50]. In our research, BBR and FLC treatment was accompanied by downregulation of the expression of the *CDR1*, *MDR*, *ALS3*, *ERG11*, and *HWP1* genes. Among them, *CDR1* and *MDR* are drug resistance genes that encode ATP-binding transporters. Researchers have shown that one of the main reasons behind FLC resistance in *Candida albicans* is the overexpression of efflux pump genes [51, 52]. Consistent with this discovery, we found downregulated expression of the *CDR1* and *MDR* genes after BBR and FLC combination treatment, indicating that BBR increased the susceptibility of *Candida albicans* to FLC in part by inhibiting drug transporter gene expression. In contrast, Marchetti et al. [53] indicated that the efflux transporter genes *CDR1*, *CDR2*, and *MDR* had independent synergistic antifungal effects with FLC against *Candida albicans*. *ALS* is one of the main gene adhesion families that controls biofilm production. In particular, the *ALS3* gene is related to biofilm formation, with its expression gradually increasing after biofilm formation [54, 55]. In our experiment, the same conclusion was obtained. Compared with FLC alone, BBR combined with FLC inhibited *ALS3* expression, which disrupted biofilm production. These results demonstrated upregulated expression of *TUP1* mRNA with filamentous growth and FLC treatment alone. *TUP1* was associated with a transcriptional repressor in the *Candida albicans*-resistant strain. Increased expression of the *TUP1* gene could inhibit the activity of hyphae after FLC and BBR combination treatment, thereby increasing drug sensitivity. *HWP1* gene production is related to the ability of cell wall proteins to stimulate a host immune response, which then causes changes at the molecular level [56]. In a previous report, the overexpression of *HWP1* significantly increased *ALS3* expression and reduced hyphal development. Consistent with this research, our results suggested that FLC had weak antifungal effects on *Candida albicans*-resistant species. Therefore, the FLC and BBR combination will be effective in reducing side effects and protecting against biofilm formation.

Ergosterol is a part of the cell membrane and has a variety of cellular functions, such as maintaining membrane integrity and membrane enzyme binding [57]. One crucial mechanism of fungal resistance to drugs is changes in the transcription of genes related to ergosterol biosynthesis. KEGG metabolic pathway analysis following whole-genome sequencing verified the previously described results regarding the effects of drugs on ergosterol biosynthesis; the sterol genes were found to be related to FLC resistance. Based on the metabolic pathway analysis, upregulated genes and other *ERG* family genes were selected for experimental testing. Of note, FLC and BBR combined treatment decreased the gene expression of *ERG1*, *ERG3*, *ERG4*, *ERG5*, *ERG24*, and *ERG25* and had a remarkably differential effect compared to single treatments. Interestingly, *ERG6* and *ERG9* expression was significantly increased compared to that in the other groups, indicating that ergosterol is closely related to the drug resistance mechanism. *ERG6* is a rate-limiting enzyme in the ergosterol biosynthesis pathway, while *ERG9* is a synthase of ergosterol and participates in feedback regulation. When combined with FLC, BBR inhibited the expression of other sterol genes, causing an increase in ERG6 and ERG9 gene expression via a feedback mechanism and accelerating the synthesis of ergosterol. In conclusion, BBR can enhance sensitivity to FLC by reducing the expression of mycelial adhesion genes, efflux-related proteins and ergosterol genes, thereby effectively reducing the hydrophobicity of the *Candida albicans* cell surface to increase sensitivity. Whole-genome sequencing can be used to identify as many resistance mechanisms as possible.

## Data Availability

All the data supporting our findings are contained within the manuscript.
